# Oro-Dental Health and Primary Nephrotic Syndrome among Vietnamese Children

**DOI:** 10.3390/children8060494

**Published:** 2021-06-10

**Authors:** Hang Minh Luong, Tra Thu Nguyen, Huy-Thinh Tran, Phung Thi Tran, Phuong-Nga Nguyen, Huong Thu Nguyen, Duc Minh Nguyen, Hanh Tran Thi Duc, Son Minh Tong

**Affiliations:** 1 School of Odonto-Stomatology, Hanoi Medical University, Hanoi 100000, Vietnam; minhhang@hmu.edu.vn (H.M.L.); tranhuythinh@hmu.edu.vn (H.-T.T.); phungtran.fsh@gmail.com (P.T.T.); nganp21@gmail.com (P.-N.N.); drmduc8@dpc.agu.ac.jp (D.M.N.); 2 Graduate School of Medicine, Nagoya University, Aichi 466-8560, Japan; 3 Health Economics, Hanoi University of Public Health, Hanoi 100000, Vietnam; 4 Nephrology and Dialysis Department, National Children’s Hospital, Hanoi 100000, Vietnam; huong141069@gmail.com; 5 Division of Research and Treatment for Oral Maxillofacial Congenital Anomalies, Aichi Gakuin University, Aichi 464-8651, Japan; 6 Department of Epidemiology, Hanoi University of Public Health, Hanoi 100000, Vietnam; ttdh@huph.edu.vn

**Keywords:** children, oral health, primary nephrotic syndrome, Vietnamese

## Abstract

Primary nephrotic syndrome (PNS) is common in children, affecting the soft and hard tissues of the oral cavity. This study aimed to investigate the percentage of dental caries, gingivitis, hypertrophic gingivitis, and developmental defects of enamel (DDE) in children with PNS. The association of PNS with these diseases and oral care behavior was also assessed. A total of 407 children with PNS and 407 age- and gender-matched controls were recruited. PNS was diagnosed based on blood and urinary tests. The Simplified Oral Hygiene Index (OHI-S), the Gingival Index (GI), the Gingival Overgrowth Index (GOI), the Decayed, Missing, and Filled Teeth Index (dmft/DMFT), and DDE were collected. The PNS patients showed significantly higher scores of OHI-S, GI, and dmft, and higher proportions of dental caries and DDE than those of the controls (*p* < 0.001). It is necessary to establish a periodic dental protocol for PNS patients to improve their oral health status.

## 1. Introduction

Nephrotic syndrome (NS) is a common chronic kidney disorder in children characterized by an excessive urinary protein level [1]. The annual incidence was reported as approximately seven per 100,000 children [2], more frequent among Asian children than among those from Europe and Africa [3]. Although NS is associated with several renal diseases, the most common form in children is primary nephrotic syndrome (PNS) [2]. PNS is frequently treated with corticosteroids and can be classified into steroid-sensitive and steroid-resistant types based on the response to therapy [1].

Drugs used in NS treatment, such as cyclosporin or nifedipine, might be a causative factor of gingivitis and hypertrophic gingivitis [4,5,6]. Hypertrophic gingivitis is typical in patients treated with cyclosporin or nifedipine [7,8]. The prevalence of gingivitis in children with PNS is from 94.3% to 100%, with mostly mild and moderate forms [8]. The hypertrophic gingivitis occurs mainly at the buccal papillae, affecting aesthetics and oral hygiene, further leading to delayed tooth eruption and malocclusion [9].

Oral hygiene practice among children with PNS has been reported as poorer than among healthy controls [10], with significantly increased food debris and dental plaque [8,11]. Despite not providing specific calculus indices, two studies by Babu and Jana (2014) [12] and Subramaniam (2012) [8] revealed a high incidence of calculus, especially at the lingual surface of mandibular incisors, in children with PNS. Routine hospitalization and a particular diet impede oral health care [13]. Evidence shows that poor oral care, high snack consumption, and the lack of parental supervision contribute to the worsening of oral health of children with PNS [10,12].

Even though poor oral care is well known as a risk factor for caries [8,12], the association between dental caries and PNS is, however, inconsistent in the literature. Several studies [7,14] revealed a significantly higher percentage of dental caries in NS children than in controls, while no difference [11,15] or contrastive results [8,10,12] were also reported.

PNS was also associated with defected development of tooth enamel (DDE) due to the disturbance of calcium and phosphate metabolism [7,11]. PNS patients may develop renal tubular acidosis, decreased urinary proton excretion, and loss of bicarbonate, leading to acid–base disorders, which affect tooth structure [16].

In Vietnam, PNS accounts for more than 90% of total children with NS [17]. However, research regarding oral health on these subjects is limited. The present study aimed to investigate (1) the percentage of dental caries, gingivitis, hypertrophic gingivitis, and DDE in children with PNS; (2) the association of PNS with these dental–oral diseases; (3) oral care behavior of children with PNS.

## 2. Materials and Methods

### 2.1. Study Participants

The study was conducted using a cross-sectional design from April 2019 to April 2020 at Vietnam National Children’s Hospital. The smallest sample size was calculated using N4studies software, version 1.4.1 for iOS. The largest group contained 262 children.

NS was diagnosed in the renal dialysis department according to the guidelines of the International Study of Kidney Diseases in Children [18,19], with the three following criteria required: proteinuria ≥ 50 mg/kg/24 h, blood protein < 56 g/L, and albumin in blood < 25 g/L. Congenital and secondary NS were ruled out by medical history, including the following: onset of NS before three months old (congenital NS) or NS present with metabolic diseases, autoimmune diseases, bee stings, poisoning, malignancies, infections, parasitic causes, or after taking certain drugs or chemical poisoning (secondary NS). Finally, 407 children with PNS were involved in the study.

Four hundred and seven controls who had no history of renal disease or systemic diseases and who were not under medication were recruited from the dental department of the hospital and matched for age and gender to the PNS group (Table 1).

Informed consent was obtained from all participants and their parents. The study was approved by the Ethics Council in Biomedical Research of Hanoi Medical University No. NCS17/HMU-IRB on 27 March 2019.

### 2.2. Oral Examination

The oral examination procedure was performed by a single pediatric dentist. We collected four indices: the Simplified Oral Hygiene Index (OHI-S), the Gingival Index (GI), the Gingival Overgrowth Index (GOI), and the Decayed, Missing, and Filled Teeth Index (dmft/DMFT). The presence of DDE was also recorded.

#### 2.2.1. Simplified Oral Hygiene Index (OHI-S)

The OHI-S was obtained by summation of the Simplified Debris Index (DI-S) and Simplified Calculus Index (CI-S) [20]. Six teeth, including four posterior and two anterior teeth, were examined for the OHI-S: 16 (55), 26 (65), 36 (75), 46 (85), 11 (51), 31 (71). Debris is the soft matter loosely attached to the teeth. It consists of mucin, bacteria, and food and varies in color from grayish-white to green or orange. The surface area covered by debris is estimated by running the side of a No. 5 explorer (Shepherd’s Crook) along the examined tooth surface. The DI-S was scored according to a five-level scale: 0—no debris or stain present; 1—soft debris covering not more than one-third of the tooth surface being examined or the presence of extrinsic stains without debris regardless of surface area covered; 2—soft debris covering more than one-third but not more than two-thirds of the exposed tooth surface, and 3—soft debris covering more than two-thirds of the exposed tooth surface.

Calculus is defined as a deposit of inorganic salts composed primarily of calcium carbonate and phosphate mixed with food debris, bacteria, and desquamated epithelial cells. There are two main types of dental calculus, which are differentiated primarily by location on the tooth in relation to the free gingival margin: (1) supragingival calculus denotes deposits usually white to yellowish-brown in color occlusal to the free gingival margin; (2) subgingival calculus denotes deposits apical to the free gingival margin. The calculus is usually light brown to black in color because of the inclusion of blood pigments. No. 5 explorers and periodontal probes were used to evaluated supragingival calculus and subgingival calculus. The CI-S was scored on a four-level scale: 0—no calculus present; 1—supragingival calculus covering not more than one-third of the exposed tooth surface; 2—supragingival calculus covering more than one-third but not more than two-thirds of the exposed tooth surface or the presence of individual flecks of subgingival calculus around the cervical portion of the tooth; 3—supragingival calculus covering more than two-thirds of the exposed tooth surface or a continuous heavy band of subgingival calculus around the cervical portion of the tooth. After the scoring of DI-S and CI-S, the scores were categorized as follows: 0–0.6 was rated as good; 0.7–1.8 was fair; and 1.9–3.0 was poor. The OHI-S score was classified as good if the score was 0–1.2, fair if the score was 1.3–3.0, and 3.1–6.0 was classified as poor oral hygiene.

#### 2.2.2. The Gingival Index (GI)

The Gingival Index is a three-level scale developed by Silness and Loe to assess the gingival tissue based on changes in gingival color and tenderness [21]. Healthy gingiva (natural coral pink; absence of inflammation, bleeding, or swelling) is coded as 0. A slight change in color and in tissue structure but without bleeding upon pressure or probing and idiopathy is coded as 1. Visible glazing, redness, edema, or hypertrophy with bleeding upon pressure or probing is coded as 2. Marked redness, hypertrophy, and a tendency towards spontaneous bleeding or ulceration is coded as 3. The GI is the average of the values for all gingival surfaces of the scored teeth. Gingivitis severity was classified as follows: 0.1–1.0: mild inflammation, 1.1–2.0: moderate inflammation, and 2.1–3.0: severe inflammation.

#### 2.2.3. The Gingival Overgrowth Index (GOI)

Gingival enlargement is assessed by measurements of overgrowth/height of the gingival tissue vertically in the apex–crown direction from the cement–enamel line to the free gingival margin. Using a periodontal probe, the dentist grades the height of the enlarged gingival covering the clinical crown and the nonvisible crown surface at six points around each tooth. GOI was coded as 0 = normal gingiva if the gingival covering <1 mm, 1 = slight if the gingival covering <2 mm and cervically equal to or less than one-third of the anatomic crown, 2 = moderate if the gingival covering was 2 to 4 mm and/or the gingiva extended to within the middle one-third of the clinical crown, and 3 = severe if the gingiva covered >4 mm and/or the gingiva covered more than two-thirds of the clinical crown [4,22].

#### 2.2.4. Decayed, Missing, and Filled Teeth (dmft/DMFT)

Dental caries were evaluated through the dmft/DMFT of the WHO [23], in which dt and DT are the number of decayed teeth, mt and MT are the number of missing teeth due to caries, and ft and FT are the number of filled teeth in primary and permanent dentition. Dental caries were diagnosed based on the International Caries Classification and Management System (ICCMS) scores, with codes 1, 2, and 3 for caries and code 0 for no caries [24]. The dmft and DMFT are obtained by the summation of dt/DT, mt/MT, and ft/FT, respectively.

#### 2.2.5. Developmental Defects of Enamel

DDE was diagnosed if the teeth met one of the following criteria: a rough or irregular enamel surface, abnormal enamel color from translucency to yellow or dark brown, or opacity enamel [25].

### 2.3. Questionnaire for Parents’ Knowledge of Oral Health and Oral Hygiene Care for Children with Systemic Diseases

We interviewed parents using a questionnaire. The questionnaire for knowledge of oral health and oral hygiene care for children with PNS was built based on the questionnaire of Steffen Koerdt et al. [26] containing 16 questions divided into three parts (Appendix A). The first part, including three questions, was for parents of children with PNS; the two other parts were for parents in two groups.

### 2.4. Statistical Analysis

The distribution of all quantity variables was checked. Differences in the percentage of gingivitis, hypertrophic gingivitis, dental caries, and DDE between PNS cases and controls were evaluated using the chi-square test. The OHI-S and dmft/DMFT indices of the two groups were analyzed with the independent samples t-test. The significance level was set at 0.05. The statistical analysis was performed with SPSS for Window version 24c (IBM, Armonk, NY, USA).

## 3. Results

### 3.1. Medication Characteristics

Among 407 children with PNS, 79 children (19.5%) were steroid-sensitive, 87 children (21.5%) were steroid-dependent, and 239 children (59%) were steroid-resistant. In the steroid-resistant group, 207 children (86.7%) were combinedly treated with cyclosporine and steroid.

### 3.2. The Percentage of Gingivitis, Gingivitis Overgrowth, Dental Caries, and DDE

Chi-square analyses show that the PNS group has a 1.301-fold higher risk of gingivitis (*p* = 0.001), a 10-times higher risk of severe gingivitis (*p* = 0.000001), and a 14.25-times higher risk of gingivitis overgrowth (*p* = 0.000001) compared with the control group. Higher percentages of dental caries and DDE among children with PNS compared with controls were also observed (odds ratio = 2.15 and 4.49, *p* = 0.0005 and 0.0003, respectively) (Table 2).

### 3.3. OHI-S and dmft/DMFT Indices

Table 3 shows the mean scores of OHI-S and dmft/DMFT in the PNS group and the control group. Significantly higher scores for OHI-S and dmft were observed in the PNS group compared with the control group (*p* = 0.0006 and 0.0008, respectively). However, DMFT was not significantly different between the two groups (*p* = 0.511).

### 3.4. Oral Health Behaviors

Most parents of children with PNS (90.7%) were not aware of the association between PNS and oral diseases. A majority (65.6%) of dentists were not aware of their children patients’ systemic disease (PNS) (Appendix A).

“Never visit dentists or visit when having problems” was the most common choice in the PNS group (49.4 %), while “visit dentists once a year” was the most common one in the control group (58.7%). Most children with PNS brushed their teeth across and fast (89.1%). On the contrary, “brush teeth circularly and slowly”, “brush teeth across and slowly”, and “brush teeth across and fast” were equally chosen (more than 30% for each) in the non-PNS group. A total of 96.8% of children with PNS never used dental floss, whereas more than half of the controls usually used dental floss (66.8%) (Appendix A).

## 4. Discussion

Our study was conducted on children who were diagnosed with primary nephrotic syndrome (PNS). The results of the study showed that the children with PNS had a higher risk of gingivitis, gingivitis overgrowth, dental caries, and DDE than the control. Additionally, the children with PNS had poorer oral hygiene status and a higher value of debris and calculus indices. Compared with the controls, the children with PNS had a higher number of carious teeth in the primary dentition. However, the number of carious teeth in the permanent dentition between the two groups was not significantly different.

The male and female group ratio was 3:1, which corresponds to the epidemiologic data of this disease [27,28]. Inconsistent with previous reports [29,30] revealing steroid dependence as forming a major group, steroid resistance in the current study accounted for 59% of total cases. The abuse and inappropriate use of steroid-containing drugs and multi-dose drug dispensing for treating common infectious diseases in developing countries seem to be contributory factors [31,32]. Moreover, we recruited PNS cases in one of Vietnam’s most prominent hospitals, which assumed a higher rate of severe and steroid-resistant patients.

### 4.1. Gingivitis and Hypertrophic Gingivitis

Corresponding to previous studies [12,33,34], our study showed a significantly higher percentage of gingivitis in the children with PNS group than in the control group. This result is consistent with poorer oral hygiene, which is reflected in the higher OHI-S of the PNS group. Additionally, the questionnaire revealed a neglect of dental checkups in the PNS group. Most children with PNS never visited dentists or visited only when having problems, never used dental floss, and brushed their teeth across and fast. This problem is understandable because these patients were frequently hospitalized for a long time; therefore, there would be less or no time for visiting dentists [10]. Other factors, such as economic hardship, lack of motivation, and stress, can also prevent them from having dental examinations [7,12]. In addition to poor oral hygiene, the reduced concentration of IgG in saliva resulting from medications for immunosuppression also leads to frequent gingivitis in children with PNS [11,15,33,35]. The majority of gingivitis cases in the PNS group were mild and moderate [7,10,12,33], possibly because most of our patients had acquired PNS for the first time and received short-term medication.

In the current study, 16.7% of children with PNS presented with hypertrophic gingivitis, while only 1.2% of controls did. The percentage of hypertrophic gingivitis among children with PNS ranged from 8% to 100% [6]. Hypertrophic gingivitis is a common side effect of cyclosporine and nifedipine, which are medications for PNS [7,10,11]. Dental plaque accumulation, the sensitivity of fibroblasts, and genetics might contribute to the disease [4].

### 4.2. Caries

In the PNS group, the average dmft of primary teeth was 5.58 ± 4.41, which was significantly higher than that of controls (4.17 ± 4.33, *p* < 0.001), suggesting children with PNS have a high risk of tooth decay. This result was similar to those of previous studies [7,14,15]. The high rate of tooth decay in children with PNS can be explained by improper attitudes towards primary dentition care, high consumption of sugar, and xerostomia, which is a side effect of steroids [7,36]. However, results to the contrary were observed in several studies, resulting from the increased concentration of urea in saliva leading to an increase in salivary pH and neutralization acids produced by bacteria [8,10,11,12]. Even though dt was high (5.21), filling score was extremely low (0.24). Without treatment, carious lesions may greatly affect the chewing force, permanent tooth germs, and the treatment outcomes of PNS [7].

Contrary to the primary dentition, the DMFT score in permanent teeth was no different between the PNS group and control group (1.36). It is commonly known that the permanent dentition is more resistant to decay than the primary one [37].

### 4.3. DDE

The percentage of DDE among children with PNS in our study was 11.1%, while the percentage in control group was only 2.9%. The children with PNS had a 4.5-fold increased risk of DDE compared with healthy peers (OR: 4.5; 95% CI: 2.3 to 8.8; *p* < 0.001). PNS patients have a disturbance of calcium and phosphate metabolism [11,38], renal tubular acidosis that decreases urinary proton excretion, and loss of bicarbonate buffer, which together affect the tooth structure [16,39,40]. A healthy diet and proper oral hygiene, such as using a very soft bristle brush and warm water, are recommended to prevent tooth decay and reduce dentin hypersensitivity [41].

### 4.4. Limitations

In the current study, the control group was collected from the dental department. The controls, therefore, were not completely healthy. However, they could still be controls since children in the dental department included first examination and routine check-up patients, as well as students from nearby schools who had routine dental examinations. A high percentage of dental floss usage in the control group could be derived from a selection bias. These subjects might have received good instruction from dentists.

## 5. Conclusions

The PNS patients, due to poor oral hygiene and drug side effects, presented high percentages of gingivitis, hypertrophic gingivitis, and dental caries. PNS was a risk factor for DDE. It is necessary to establish a periodic dental protocol for PNS patients to improve their oral health status.

## Figures and Tables

**Table 1:** Participants distributed by gender and age.

Group	Gender		Age		
	Male	Female	3–6	7–12	13–18
PNS	309	98	123	210	74
Control	309	98	123	210	74
Total	618	196	246	420	148

PNS: Primary nephrotic syndrome.

**Table 2:** The percentage of oral diseases in PNS and non-PNS groups.

Oral Diseases	PNS (*n* (%))	Non-PNS (*n* (%))	Odds Ratio	95% Confidence Interval	*p*-Value
Gingivitis	281 (79.0%)	203 (49.9%)	1.301	1.112–1.523	0.001
Mild	209 (51.3%)	139 (34.1%)			
Moderate	57 (14.0%)	64 (15.8%)			
Severe	15 (3.7%)	0 (0%)	10	3.611–27.693	0.000001
Gingivitis overgrowth	68 (16.7%)	5 (1.2%)	14.25	5.219–38.908	0.000001
Mild	48 (11.8%)	4 (1.0%)			
Moderate	17 (4.2%)	1 (0.2%)			
Severe	3 (0.7%)	0 (0%)	0.992	0.984–1.001	0.249
Dental caries	320 (78.6%)	257 (63.1%)	2.15	1.57–2.93	0.0005
DDE	45 (11.1%)	11 (2.7%)	4.49	2.29–8.81	0.0003

DDE: developmental defects of enamel.

**Table 3:** Comparison of OHI-S, GI, GOI, and dmft/DMFT indices between PNS and non-PNS groups.

Variables	PNS		Non-PNS		*p*-Value
	Mean	SD	Mean	SD	
*DI-S*	1.22	0.66	0.84	0.71	0.0006
*CI-S*	0.53	0.76	0.28	0.45	0.0007
OHI-S	1.75	1.20	1.13	1.04	0.0006
*dt*	5.21	4.32	3.63	4.07	0.0006
*mt*	0.14	0.53	0.14	0.78	0.285
*ft*	0.24	0.81	0.40	1.25	0.845
dmft	5.58	4.41	4.17	4.33	0.0008
*DT*	1.26	1.75	1.54	2.23	0.721
*MT*	0.02	0.18	0.01	0.13	0.449
*FT*	0.08	0.49	0.16	0.65	0.09
DMFT	1.36	1.89	1.71	2.36	0.511

DI-S: Simplified Debris Index; CI-S: Simplified Calculus Index (CI-S); OHI-S: Simplified Oral Hygiene Index; DT/dt: decayed teeth; MT/mt: missing teeth; FT/ft: filled teeth; DMFT/dmft: Decayed, Missing, and Filled Teeth.

## Data Availability

The data that support the findings of this study are available from the corresponding authors (T.T.N. and S.M.T.) upon reasonable request.

## Appendix A

**Variables**	**PNS (*N* = 407)**	**Non-PNS (*N* = 407)**
1. At the first diagnosis of NPS of your child, did the doctor mention related issues of oral health? *n* (%)		
No	369 (90.7%)	-
Yes	38 (9.3%)	-
2. Do you recognize the relationship between oral health and PNS? *n* (%)		
No	367 (90.2%)	-
Yes, but not fully informed	33 (8.1%)	-
Yes, fully informed	7 (1.7%)	-
3. Did the dentist of your child recognize his/her NPS? *n* (%)		
No	143 (65.6%)	-
Yes, but he did not mention anything else	56 (25.7%)	-
Yes, and he reminded us of some issues	19 (8.7%)	-
4. How often does your child visit a dentist? *n* (%)		
Never or when having problems	201 (49.4)	26 (6.4)
Less than once/year	102 (25.1)	28 (6.9)
Once/year	51 (12.5)	239 (58.7)
2 times/year	35 (8.6)	89 (21.9)
More than 2 times/year	18 (4.4)	25 (6.1)
5. How often does your child brush his/her teeth? *n* (%)		
Never	13 (3.2)	1 (0.3)
Once/day	167 (41.0)	82 (20.1)
Twice/day	222 (54.6)	320 (78.6)
More than 2 times/day	5 (1.2)	4 (1.0)
6. Does your child have a toothbrush of his/her own? *n* (%)		
No	4 (1.0)	1 (0.25)
Yes, a hard one	18 (4.5)	64 (15.0)
Yes, a soft one	377 (93.5)	344 (84.5)
Do not remember/Do not know	4 (1.0)	1 (0.25)
7. How long does it take whenever your child brushes their teeth? *n* (%)		
Less than 3 min	366 (90.8)	352 (88.0)
3–5 min	5 (1.3)	27 (6.8)
Do not remember/Do not know	32 (7.9)	21 (5.2)
8. How does your child brush her/his teeth every day? *n* (%)		
Circular and Slow	26 (6.5)	126 (31.0)
Top–bottom and Slow	18 (4.5)	127 (31.3)
Across and Fast	358 (89.1)	152 (37.4)
Across and Slow	0	1 (0.3)
9. How often do you check the dental hygiene of your child? *n* (%)		
Never	277 (68.2)	192 (47.2)
1–3 times/week	78 (18.7)	159 (39.1)
Daily	53 (13.1)	56 (13.7)
10. How often does your child use dental floss? *n* (%)		
Never	394 (96.7)	135 (33.2)
Once/month	5 (1.2)	38 (9.3)
Once/week	2 (0.5)	33 (8.1)
Once/day	3 (0.8)	98 (24.1)
Every time	3 (0.8)	103 (25.3)
11. Does your child have fluoride supplements? *n* (%)		
No	42 (10.3)	10 (2.5)
Yes, with fluoride in toothpaste	359 (88.2)	390 (95.8)
Yes, with fluoride salt	5 (1.2)	3 (0.7)
Yes, with fluoride oral pill	0	2 (0.5)
12. Has your child ever had toothaches? *n* (%)		
No	191 (46.9)	225 (55.3)
Yes	209 (51.4)	139 (34.2)
Do not remember/Do not know	7 (1.7)	43 (10.6)
13. How often do you note gum bleeding? *n* (%)		
Never	234 (57.6)	270 (66.3)
1/week	83 (20.4)	41 (10.1)
1/day	42 (10.3)	59 (14.5)
Every time	8 (2.0)	4 (1.0)
Do not remember/Do not know	39 (9.6)	33 (8.1)
14. How often does your child eat sweets? *n* (%)		
Never	70 (17.2)	7 (1.7)
1/day	79 (19.4)	68 (16.7)
2/day	135 (33.2)	143 (35.1)
3/day	102 (25.1)	84 (20.6)
More often/day	19 (4.7)	104 (25.6)
Do not remember/Do not know	2 (0.5)	1 (0.3)
15. How many sweetened beverages does your child consume? *n* (%)		
None	112 (27.5)	30 (7.4)
Less than 0.5 L/day	229 (56.3)	253 (62.2)
0.5–1 L/day	63 (15.5)	114 (28.0)
More than 1 L/day	2 (0.5)	3 (0.7)
Do not remember/Do not know	1 (0.3)	7 (1.7)
16. How often did your child consume sweetened tea, lemonade, or juices during his/her early childhood (aged 0–4)? *n* (%)		
Never	69 (16.9)	16 (3.9)
1/week	72 (17.7)	50 (12.3)
1/day	117 (28.7)	129 (31.7)
2/day	91 (22.4)	92 (22.6)
More often	21 (5.2)	99 (24.3)
Do not remember/Do not know	37 (9.1)	21 (5.2)
